# Medical and Physical Intelligence

**Published:** 1821-05

**Authors:** 


### Medical and Physical Intelligence.

ALTHOUGH it is not a proper subject for a *critical analysis*, we are disposed to make a few remarks, in this place, on a new translation of the Pharmacopœia of the Royal College of Physicians of London, that has been produced by Mr. Collier.* "Having for some years been employed in instructing medical *Tyros* in the Latin language," (Mr. Collier says, in his Preface to the work just alluded to,) "and supplying those deficiencies in chemistry and botany which form the *sine qua non* to their passing their examination before the Court of Examiners at Apothecaries' Hall, it was natural for me to inquire by what means I could most easily accomplish my task, to my own credit and that of my pupils. Since the passing of the Act of Parliament 53d of Geo. III. entitled 'An Act for the better regulating the Practice of Apothecaries throughout England and Wales,' something like a classical education seems indispensably necessary for pupils, if they would go through their examination with credit to themselves. Yet has it been a standing complaint with that 'honourable and intelligent body,' that many appear before them for examination who are wholly ignorant of the Latin language and the common rudiments of chemistry. It is in consequence of these deficiencies that I have had so many young men under my care; and it has been my labour to give them such a competent knowledge of the language as should enable them to translate physicians' prescriptions and the Pharmacopœia of the Royal College of Physicians. Now, to effect this in a short time, I knew no better method than to put into their hands a literal translation of that work; and, not being able to find any, I naturally enough set about the business myself, and have endeavoured to give it them in that state which I hope will answer their purpose and mine." Mr. Collier has also annexed to each preparation, in which chemical operations are concerned, a theory of those operations, which, he thinks, "will give them (alluding here to "private practitioners" and druggists,) that succinct idea of the chemical decompositions, as shall enable them to avoid many errors."

On the value of such a mode and extent of instruction, as well as the fact that Mr. Collier has "hitherto instructed near a hundred pupils in the same plan, and never yet had one rejected from the Hall," it is not our business to make any critical remarks;—the history of no small proportion of the Theses produced at some of the most celebrated Universities, as we all know, need not make students thus *ground* very much dissatisfied with themselves:—but it is proper that we should say something respecting the manner in which Mr. Collier has fulfilled his intentions. The translation is, with a few exceptions,† as strictly verbal as it is well possible to render it, at the

* Published by Highley and Son, 1821.

† As, in the "Preface of the College," (for example,) in the concluding sentence of the second paragraph, where "*always*" is introduced;—in the next paragraph, "*so well*" is superfluous,—and "*their* sanction and authority," where same time that it is not remote from some degree of neatness of expression: a specimen of success requiring a greater command of language than those who have not attempted such a task may probably imagine. Mr. Collier's theories of the chemical processes are correct, in conformity with the established principles of chemistry. In the few obscure examples of chemical operation which the Pharmacopœia contains, (as those in the preparation of tartarized antimony and antimonial powder,) he agrees with the best chemists, excepting on one point, that of the composition of the black sulphuret of mercury; where he does not appear to be informed of the observations of GUIBOURT, who has shown it to be constituted, not of a uniform sulphuret of mercury, but of the red sulphuret, or cinnabar, mingled with merely minutely-divided metallic mercury. This part of the work would have been more useful to students than it is at present, had the author comprised, in his own descriptions, the names of the several compounds used in the new chemical nomenclature: for example, when objecting to the term *sub*-muriate of mercury (applied to calomel), and stating that it is a muriate at the minimum of oxydizement, he might have added, or a *proto*-chloride; that the oxymuriate is a *deuto*-chloride, and so of the rest. This would give them more precise ideas, and at the same time enable them to understand such terms when they may meet with them in the medical writings of other nations. As it is, the notes form an addition to the translation that should obtain for it the preference to the others by persons who are not themselves well versed in chemistry,—without paying any regard to the fulfilment of the author's express intentions of rendering the work more peculiarly useful to any particular class of students.

---

As the extent to which the space of this Number of the Journal is occupied by articles of Review which we have been unable to render more concise, still prevents our fulfilling our promise to give an abstract of the whole of the papers in the first Number of Dr. MAGENDIE'S Journal for Experimental Physiology, we shall at present produce only an account of the memoir *on the Mechanism of Absorption in Red and Warm-blooded Animals*, by Dr. MAGENDIE; and complete our abstract at future periods, as the arrangements of the Journal may permit.

The memoir commences with a demonstration of the fact that ab-

---

the pronoun is without an antecedent; at the same time that the version is not quite correct, any more than that, in the next paragraph, of "ea tandem et stabilia maximè et utilia fore;—where "medicos" is translated "*practitioners;*"—where "*Satius* igitur esse putavimus" is translated "we therefore thought it *wiser;*"—where, alluding to the division of the gallon into parts, "pro arbitrio" is omitted;—and where "suavissima" is translated "most *sweet:*" with a few others, in different parts of the work, of no further import than that they are deviations from a "literal" translation; amongst which there is, however, one (which frequently occurs,) that we should notice, because it would indicate a grammatical fault in the composition of the original which does not exist: it is where Mr. Collier translates "sepone, ut fiant crystalli," "set it aside, that it may *crystallize,*" and then says, "dry *these (crystals)*, &c." Here the pronoun and the noun are without a relative antecedent,—from his using the verb *crystallize* instead of the noun *crystals*,—which is, as it is obvious, not the case in the original.

sorption is effected in animals. Having proved the fact, the next point is to determine the agents of this function. Dr. Magendie says, that the results of experiments related in his former memoirs on this subject shew that—

"1°. The sanguiferous veins are endowed with the absorbent faculty.

"2°. It is not demonstrated that the vessels which absorb the chyle can absorb other matters.

"3°. The absorbent power of such of the lymphatic vessels as are not chyliferous, is not yet established on sufficiently satisfactory evidence."

Then follow remarks that these inferences are qualified to explain how it is that substances are absorbed in parts devoid of lymphatic vessels, (as Dr. Magendie thinks proper to say,) as "the brain, the eye, &c.;" and an attempt to ridicule the notions that the supposed absorbent vessels exert a sort of "discernment" in the selection of the fluids they transmit, (which ridicule is just as well applied here as it would be to the notion that the larynx has a sort of "discernment," since it admits air and refuses to admit brandy; or that the stomach possesses such a faculty, since it retains and digests a potato, and rejects a root of ipecacuanha.)

Dr. Magendie then states that his experiments have shown that, when a certain quantity of warm water has been injected into the veins of an animal, absorption is impeded; and when the quantity of blood is diminished, by abstraction, absorption is accelerated; and that, when a certain quantity of blood is abstracted, and replaced by the same quantity of warm water, absorption is effected as rapidly as it is under ordinary circumstances. These facts have led the author to infer that absorption is a purely physical action in the animal economy, and depends on "the capillary attraction of the vascular parietes for the matters absorbed." After having adduced some considerations which he thinks favour this explanation, he proceeds to relate the results of experiments which he believes prove it to be true. This explanation, he remarks, would indicate that absorption should be effected by vessels after death, as well as during life; and this he considers as also proved by his experiments. Dr. Magendie says—

"I took a portion of the external jugular vein of a dog: (this portion of vessel, in length nearly an inch, received no collateral branch:) I deprived it of surrounding cellular tissue; I attached to each of its ends a glass tube, by means of which I established a current of warm water in its interior. I then plunged the vein in a slightly-acid liquor, and I carefully collected the fluid which formed the interior current.

"It is evident, from the disposition of the apparatus, that there could be no communication between the interior current of warm water and the exterior acid liquor.

"For the first few minutes the liquor which I collected had suffered no change; but, after five or six minutes, the water became sensibly acid. Absorption had then taken place.

"I repeated this experiment on veins taken from dead human bodies: the effect was the same."

Similar results were obtained from similar experiments with a portion of an artery, when it was besides remarked that, "the more acid the external fluid, and the more high the temperature, (below a certain distance from the boiling point,) the more rapidly the phenomenon was produced."—"If capillary absorption occurs in large dead blood-vessels, why should it not take place in similar living vessels?" Aware of the obvious reply of a sceptic, Dr. Magendie put this question to the test of experiment. He says—

"I took a dog about six weeks old, at which age the vascular parietes are thin, and consequently particularly calculated for the success of the experiment. I laid bare one of the jugular veins, isolated it completely throughout its whole length; carefully separated it from the parts adjacent, especially the cellular tissue and some small vessels which ramified from it; and placed a card beneath it, in order that the vessel might have no contact with the surrounding part. I then let fall on its surface, at the middle of the card, a thick aqueous solution of an alcoholic extract of nux vomica, a substance the action of which on dogs is very energetic: I took care that no portion of the poison should touch any other part than the vein and the card, and that the course of the blood was free in the interior of the vessel. Before the lapse of the fourth minute, the effects which I expected were manifested, at first but feebly, but afterwards with such severity that insufflation of the lungs was necessary for the preservation of the life of the animal."

Similar results were obtained, on repeating the experiment on an adult dog. It remained to try it on an artery. This was effected on the carotid arteries of two large rabbits. The effects were developed in both animals, and one of them died. "To assure myself," Dr. Magendie says, "that the poison had really traversed the parietes of the artery, and that it had not been absorbed by the small veins which might have evaded my dissection, I carefully detached the vessel which had been the subject of the experiment; I divided it throughout its length; and I persuaded the persons who assisted me to taste the little blood which had remained adherent to its interior surface: they all recognized, as well as myself, the extreme bitterness of the extract of nux vomica."

In order to ascertain whether the minute vessels also permitted fluids to traverse their parietes, Dr. Magendie made the following experiment:—"I took," he says, "the heart of a dog who had died on the preceding evening; I injected into one of its coronary arteries, water of 30° centig. (85° Fahren.) This water readily returned by the coronary vein into the right auricle, whence it flowed into a vessel. I poured into the pericardium half an ounce of water slightly acidulated. At first the injected water showed no sign of acidity; but, on the lapse of five or six minutes, it presented unequivocal evidence of the possession of that quality." Thus, the fact in question appeared to be proved in respect to dead minute vessels: in regard to living vessels of this class, Dr. Magendie says, "it was not necessary to have recourse to new inquiries, nor to sacrifice more animals. The experiments which I mentioned in my memoir on *the Organs of Absorption in the Mammalia*, left no doubt on this point, according to the judgment of the Academy."

In respect to the permeability of membranes by certain fluids during life, (evidence of which after death we have in the colouring of the parts adjacent to the gall-bladder with bile,) Dr. Magendie observes, that he has often seen membranes penetrated and coloured by substances placed in contact with them: for example, if a certain quantity of ink is introduced in the cavity of the pleura of a young dog, the period of hardly an hour is requisite in order that the pleura, the pericardium, the intercostal muscles, and the surface of the heart itself, may be rendered black.

Amongst his inferences from the foregoing facts, Dr. Magendie remarks, that "the capillary attraction of the parietes of the small vessels appears to be the cause, or, more exactly, one of the causes, of the absorption called venous;" whilst he takes care to add, that "this conclusion does not 'touch' in any respect the absorption which is effected in the small intestine on the chyle, by the chyliferous vessels, and still less the absorbent property of the lymphatics; though the experiments I have just described seem to indicate, that if, in the greater number of instances, these vessels do not absorb, this depends not on their parietes, which have properties similar to those of the veins, but to the want of a continuous current in their interior."

---

### BIOGRAPHICAL NOTICE OF THE LATE DR. GREGORY.*

### METEOROLOGICAL JOURNAL.

By Messrs. William Harris and Co. 50, Holborn, London.

*From March* 20 *to April* 19, *inclusive.*

| Day of Month. | Moon. | Rain gauge | THERM. | | | BAROM. | | DeLuc's Hygro. | | WIND. | | ATMOSPHERIC VARIATION. | | |
|---|---|---|---|---|---|---|---|---|---|---|---|---|---|---|
| Mar 20 | | | 39 | 50 | 38 | 29·30 | 29·30 | 53 | 53 | W | WNW | Fine | | Cloud. |
| 21 | | | 40 | 50 | 34 | 29·26 | 29·50 | 55 | 55 | W | NW | Fine | | |
| 22 | | ·09 | 38 | 47 | 32 | 29·66 | 30·00 | 55 | 56 | NNW | N | Fine | Sho'ry | Fine |
| 23 | | | 34 | 45 | 33 | 30·06 | 30·00 | 54 | 54 | N | SSW | Fine | | |
| 24 | | ·20 | 37 | 49 | 35 | 30·00 | 29·85 | 53 | 57 | SW | SW | Fine | Sho'ry | Rain |
| 25 | | ·13 | 39 | 49 | 36 | 29·73 | 29·60 | 60 | 59 | SW | W | Rain | | Fine |
| 26 | ☽ | | 38 | 51 | 36 | 29·51 | 29·45 | 56 | 55 | SW | S | Fine | | Cloud. |
| 27 | | | 41 | 50 | 38 | 29·40 | 29·41 | 55 | 58 | SW | SW | Fine | | |
| 28 | | ·16 | 40 | 53 | 40 | 29·27 | 29·15 | 60 | 60 | SSE | SE | Cloudy | Rain | |
| 29 | | ·29 | 41 | 52 | 38 | 29·23 | 29·37 | 60 | 58 | S | SW | Rain | | |
| 30 | | | 41 | 49 | 40 | 29·70 | 29·72 | 58 | 58 | WSW | SSW | Fine | | |
| 31 | | ·27 | 42 | 48 | 36 | 29·43 | 29·57 | 58 | 57 | S | W | Rain | Sho | Fine |
| Apr 1 | | ·11 | 39 | 50 | 47 | 29·66 | 29·53 | 58 | 58 | WNW | SSE | Fine | | Rain |
| 2 | ● | ·21 | 49 | 53 | 45 | 29·33 | 29·33 | 63 | 62 | W | W | Rain | | |
| 3 | | | 47 | 55 | 47 | 29·33 | 29·35 | 60 | 55 | SW | SW | Fine | | |
| 4 | | | 45 | 57 | 49 | 29·38 | 29·41 | 57 | 57 | SW | WNW | Fine | | |
| 5 | | | 45 | 53 | 43 | 29·78 | 29·90 | 55 | 56 | N | NNW | Fine | | |
| 6 | | ·19 | 47 | 51 | 42 | 29·95 | 29·99 | 57 | 59 | N | N | Fine | | Rain |
| 7 | | | 45 | 55 | 49 | 30·06 | 30·17 | 63 | 62 | N | W | Cloudy | | |
| 8 | | | 52 | 60 | 51 | 30·16 | 30·13 | 60 | 60 | NW | W | Fine | | |
| 9 | ☾ | | 55 | 61 | 52 | 30·05 | 29·93 | 58 | 58 | WNW | WNW | Fine | | Cloud. |
| 10 | | | 56 | 60 | 48 | 29·82 | 29·74 | 57 | 60 | W | SW | Fine | Cloud. | |
| 11 | | ·24 | 53 | 59 | 42 | 29·67 | 29·54 | 55 | 57 | SW | SSW | Showery | Fine | Rain |
| 12 | | ·10 | 46 | 53 | 44 | 29·45 | 29·40 | 55 | 57 | WSW | SW | Fine | Sho'ry | Colud. |
| 13 | | | 47 | 52 | 41 | 29·37 | 29·71 | 56 | 56 | W | W | Fine | | |
| 14 | | ·13 | 45 | 59 | 40 | 29·67 | 29·43 | 55 | 56 | SSE | S | Fine | Rain | Cloud. |
| 15 | | ·18 | 43 | 50 | 38 | 29·55 | 29·54 | 55 | 56 | SW | S | Rain | Sho'ry | Fine |
| 16 | | | 42 | 52 | 41 | 29·51 | 29·65 | 56 | 60 | S | S | Fine | | |
| 17 | ○ | | 43 | 55 | 39 | 29·77 | 30·00 | 57 | 60 | SSE | S | Fine | | |
| 18 | | | 42 | 54 | 44 | 29·82 | 29·60 | 57 | 56 | SE | ESE | Fine | | |
| 19 | | ·04 | 50 | 59 | 43 | 29·51 | 29·49 | 58 | 60 | E | E | Cloudy | | |

The quantity of rain fallen in the month of March, is 2 inches and 64-100ths.

